# Design of the Pregnancy REmote MOnitoring II study (PREMOM II): a multicenter, randomized controlled trial of remote monitoring for gestational hypertensive disorders

**DOI:** 10.1186/s12884-020-03291-2

**Published:** 2020-10-15

**Authors:** Dorien Lanssens, Inge M. Thijs, Wilfried Gyselaers, PREMOM II – consortium, Wilfried Gyselaers, Inge M. Thijs, Dorien Lanssens, Eric T. De Jonge, Caroline Van Holsbeke, Tinne Mesens, Yves Jacquemyn, Dominique Mannaerts, Roland Devlieger, Kristel Van Calsteren, Hilde Logghe, Barbara Lebbe

**Affiliations:** 1 Ziekenhuis Oost-Limburg, Future Health Department, Limburg Clinical Research Center/Mobile Health Unit, Genk, Belgium; 2 Department of Obstetrics and Gynaecology, Ziekenhuis Oost-Limburg, Genk, Belgium; 3 Faculty of Medicine and Life Sciences, Limburg Clinical Research Center/Mobile Health, UnitUHasselt – ZOL, Hasselt University, Martelarenlaan 42, 3500 Hasselt, Belgium

**Keywords:** Remote monitoring, Gestational hypertensive disorders, Pre-eclampsia

## Abstract

**Background:**

Observational data from the retrospective, non-randomized Pregnancy REmote MOnitoring I (PREMOM I) study showed that remote monitoring (RM) may be beneficial for prenatal observation of women at risk for gestational hypertensive disorders (GHD) in terms of clinical outcomes, health economics, and stakeholder perceptions. PREMOM II is a prospective, randomized, multicenter follow-up study that was performed to explore these promising results.

**Methods:**

After providing written consent, 3922 pregnant women aged ≥18 years who are at increased risk of developing GHD will be randomized (1:1:1 ratio) to (a) conventional care (control group), (b) a patient self-monitoring group, and (c) a midwife-assisted RM group. The women in each group will be further divided (1:1 ratio) to evaluate the outcomes of targeted or non-targeted (conventional) antihypertensive medication. Women will be recruited in five hospitals in Flanders, Belgium: Ziekenhuis Oost-Limburg, Universitaire Ziekenhuis Antwerpen, Universitaire Ziekenhuis Leuven, AZ Sint Jan Brugge–Oostende, and AZ Sint Lucas Brugge. The primary outcomes are: (1) numbers and types of prenatal visits; (2) maternal outcomes; (3) neonatal outcomes; (4) the applicability and performance of RM; and (5) compliance with RM and self-monitoring. The secondary outcomes are: (1) cost-effectiveness and willingness to pay; (2) patient-reported outcome measures (PROMS) questionnaires on the experiences of the participants; and (3) the maternal and perinatal outcomes according to the type of antihypertensive medication. Demographic, and maternal and neonatal outcomes are collected from the patients’ electronic records. Blood pressure and compliance rate will be obtained from an online digital coordination platform for remote data handling. Information about the healthcare-related costs will be obtained from the National Coordination Committee of Belgian Health Insurance Companies (Intermutualistisch Agentschap). PROMS will be assessed using validated questionnaires.

**Discussion:**

To our knowledge, this is the first randomized trial comparing midwife-assisted RM and self-monitoring of prenatal blood pressure versus conventional management among women at increased risk of GHD. Positive results of this study may lead to a practical framework for caregivers, hospital management, and payers to introduce RM into the prenatal care programs of high-risk pregnancies.

**Trial registration:**

This study was registered on clinicaltrials.gov, identification number NCT04031430. Registered 24 July 2019, https://clinicaltrials.gov/ct2/show/NCT04031430?cond=premom+ii&draw=2&rank=1.

## Background

Worldwide, 5–8% of pregnant women develop gestational hypertensive disorders (GHD). In Flanders and Universitaire Ziekenhuis Brussels, the prevalence of GHD was 4.6% [1]. This means that ca. 3000 of 64,000 pregnancies in Flanders are complicated with this disorder each year. Of these, ca. 200 women (6.6%) delivered before the gestational age of 34 weeks because of GHD, 400 (13.3%) delivered between 34 and 37 weeks, and 2400 (80.1%) delivered after 37 weeks.

There are three main types of hypertension in pregnancy: essential/chronic hypertension (EH); gestational hypertension (GH); and pre-eclampsia (PE) [2]. EH is defined as a high blood pressure (> 140/90 mmHg, measured twice with an interval of 6 h), detected before conception or that develops during the first 20 weeks of gestation. This condition is associated with PE, intra-uterine growth restriction, and placental abruption [3–5]. GH is defined as elevated blood pressure (> 140/90 mmHg, measured twice with an interval of 6 h) that occurs after 20 weeks of gestation. Approximately 50% of all women diagnosed with GH will develop PE between 24 and 35 weeks of pregnancy [6]. PE involves hypertension accompanied by protein loss (> 300 mg/24 h) [2, 7], and is classified as early PE if it is diagnosed before 34 weeks of gestation, or as late PE if diagnosed after this time [8]. Recent definitions of PE also include maternal organ failure (e.g., renal insufficiency, liver disorders, neurological disorders, or hematological complications), utero placental dysfunction, or fetal growth retardation. If left untreated, PE is often fatal and, in low-income countries, it is a major cause of maternal and fetal mortality [9].

Women with an elevated risk of developing PE are more intensively followed up than women with an uncomplicated pregnancy. This results in an increased number of prenatal consultations and, when necessary, hospitalization to a prenatal ward for observation of the mother and the unborn child in order to regulate the medication schedule or to induce the delivery. Although randomized controlled trials of pregnant women at risk of developing GHD have shown that appropriate blood pressure measurement is an important component of prenatal care, standardized care processes that enable routine blood pressure measurement have not yet been introduced in clinical practice [10].

### Pregnancy REmote MOnitoring I study (PREMOM I)

**Fig. 1:**
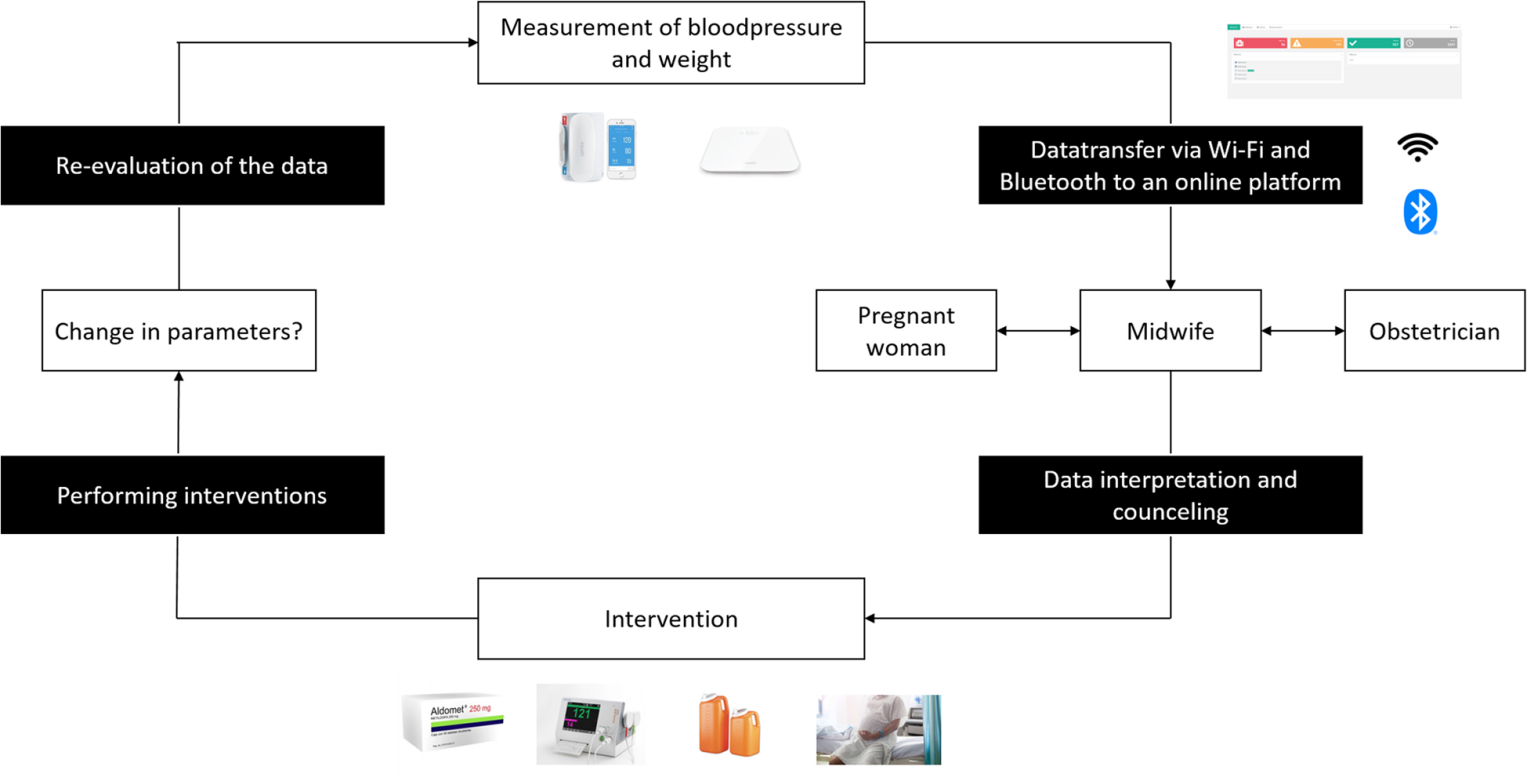
Workflow of PREMOM I

By adding remote monitoring (RM) to the current prenatal follow-up program, it is possible to address the shortcoming described above. RM can be defined as the use of telecommunication technologies to assist the transmission of medical information between the patient and the caregiver [11]. This is a relatively new technique (it was used for the first time in the ‘90s) that facilitates home-based management of patients [12]. The PREMOM I study was set up in January 2015 as a collaboration in Belgium between Hasselt University, Ziekenhuis Oost-Limburg (ZOL; Genk), and seven other hospitals (AZ Vesalius, Tongeren; Heilig Hart Ziekenhuis, Mol; JESSA, Hasselt; Maria Ziekenhuis Noord Limburg, Overpelt; Sint Franciskusziekenhuis, Heusden; S. Trudo, Sint Truiden; and Ziekenhuis Maas & Kempen, Maasmechelen). Women who participated in the PREMOM I study received obstetric surveillance via a blood pressure monitor, an activity tracker, and a weight scale. They were asked to perform two blood pressure measurements each day (morning and evening), to wear the activity tracker continuously, and to register their weight once a week in the app. This information was to be recorded until the moment of their delivery or until they were admitted to the hospital. The data collected using these devices were transferred to an online dashboard developed by the Mobile Health Unit (Limburg Clinical Research Center, Hasselt University – ZOL – Jessa). Predefined alarm signals were developed. Alarm signals were communicated to the responsible obstetrician so that treatment options could be agreed with the midwife before the pregnant woman was contacted. The therapeutic interventions were in line with local treatment procedures. The workflow is summarized in Fig. 1.

## RM for pregnancies complicated with GHD

### Observational studies and clinical outcomes

In the last few years, our research team has published two articles related to the PREMOM I project that demonstrated the benefit of RM for pregnant women at elevated risk of developing GHD [13, 14]. Both articles made comparisons between women who received RM and women who had an increased risk of developing GHD but did not participate in the PREMOM I study (conventional care, control group [CG]) based on a retrospective and observational design. In both studies, prenatal hospitalization to a prenatal ward (until the moment of delivery), diagnosis of PE, and number of inductions were reduced in the RM group compared with the CG. However, women in the RM group had significantly higher risks of developing GH and spontaneous start of the birth process compared with the CG. In the study conducted in 2015, the total number of neonatal hospitalizations to the neonatal intensive care unit (NICU) was lower in the RM group than in the CG group; these findings were not confirmed in the study conducted in 2015–2016. In the later study, the total number of prenatal visits was lower in the RM group than in the CG group; this difference was not apparent in the earlier study [13, 14].

To our knowledge, there have been no published articles describing the effects of RM for prenatal follow-up of women at risk for GHD since the prior PREMOM I studies. Nevertheless, numerous studies have investigated the feasibility of connected apps and digital devices for women at increased risk of pregnancy complications [15]. Many studies have shown that RM and home blood pressure measurements are a reliable and feasible manner to manage blood pressure in non-pregnant patients with hypertensive disorders [16–20]. Some studies have also compared usual care and RM for the management of postpartum hypertension, and concluded that RM is more successful than usual care in the follow-up and monitoring of blood pressure, and to detect warning signals [21, 22].

### Health economic considerations

The PREMOM I study also considered the health economics of RM for pregnant women in the 2015 and 2016 groups. RM enabled an average cost reduction for the Belgian National Health Care System of €740.39 per pregnancy (14.89%; 2015 data) up to €1950.37 per pregnancy (2015 + 2016 data). This cost saving was due to the reduction in prenatal visits, prenatal admissions, and neonatal admissions to the NICU [23, 24]. The savings were mostly related to neonatal care, particularly for neonates born at a gestational age of < 34 weeks, with a total cost reduction of €9123.16 per pregnancy in the RM group relative to the CG group. The cost reduction per pregnancy in the RM group versus the CG group was €225.86 for neonates born between 34 and 37 weeks and €35.93 for neonates born after 37 weeks. The finding that the main costs savings are driven by reduced neonatal care is not surprising since neonatal care is particularly intensive and is one of the most expensive forms of in-hospital care [25]. GHD-related neonatal morbidities include complications of prematurity, and the severity of prematurity is correlated with the cost of neonatal care [26]. Since the publication of those articles, we are not aware of additional health economic analyses of RM in pregnancy. Furthermore, there are few reports covering similar topics in non-pregnant patients.

### Experiences of the stakeholders

As part of the PREMOM I study, we examined the perceptions of midwives, obstetricians, and pregnant women, and found that the majority of healthcare professionals had none or little experience of RM before the study started [27]. However, after 1 year of using RM, the healthcare professionals felt that RM is an important tool in the prenatal follow-up of women at increased risk of developing GHD. They would also recommend it to their colleagues, and would roll-out this program to other centers in Belgium offering prenatal follow-up of pregnant women at increased risk of GHD. The pregnant women also reported a feeling of safety during their pregnancy and that they had no concerns about sharing their health status with the healthcare professionals. Most of the women wanted to be contacted, preferably by telephone, within 3–12 h after recording abnormal values. This implies the importance of 24/7 surveillance of the vital parameters of pregnant women at increased risk of developing GHD [27]. The compliance rate for blood pressure measurement was also high for morning (89.16%) and evening (89.00%) [27]. To our knowledge, there are no other articles describing the perceptions of stakeholders to RM of women at increased risk of developing GHD.

## Methods/design

### Aim of PREMOM II

While the PREMOM I study yielded positive and promising results, there are some aspects that warrant further investigation.

- Increasing the external validity via a multicenter, randomized design.
- Performing thorough analyses of the factors that contribute to the benefit of RM for GHD, because blood pressure measurement itself does not have a specific effect. The research team assumes that the benefit of RM involves supervision from a midwife with knowledge of normal pregnancies and pathological events, and can anticipate potential clinical events when necessary. To investigate this, the timing and type of interventions will be meticulously registered.
- An uncontrolled form of RM will be built in, which totally relies on the motivation of the pregnant women. This group would be the patient self-monitoring (PSM) group, and will be included in PREMOM II.

PREMOM II is a prospective, multicenter, randomized controlled trial that aims to assess the benefit of RM for prenatal follow-up of pregnant women at increased risk of developing GHD.

### Study design

PREMOM II is a multicenter, randomized controlled trial that will be performed at five hospitals with their own prenatal wards between October 1, 2018 and March 30, 2022. The centers combined will perform more than 7000 deliveries per year. Eligible participants will be pregnant women ≥18 years old, who are able to understand oral and written information, with a minimum risk of 1/100 on the Fetal Medicine Foundation Tool (FMF) [28, 29]. By using the FMF tool will a risk assessment for the development of early PE be made, in which the following parameters are taken into account: (1) maternal characteristics; (2) medical history; (3) obstetric history; (4) information about the current pregnancy; (5) biophysical measurements. Women will be excluded if a congenital malformation is detected or if they do not have a smartphone.

The ethics committee at Universitair Ziekenhuis Antwerpen (UZA; Wilrijk) approved the study in July 2019 (Belgium Registration Number: BE300201938651). The local ethics committees at ZOL, Universiteit Ziekenhuis Leuven (Leuven), AZ Sint Jan (Bruges), and AZ Sint Lucas (Bruges) provided advice to the ethics committee at UZA before the study was approved.

General information about the study will be available at the antenatal care units at each site. All women who are pregnant for the first time, or who had a history of GHD in previous pregnancies, are given oral and written information about the study when they undergo their first trimester ultrasound examination. They will also be asked whether they want to be screened for the development of early PE. Women who are interested in participating the study are asked to give their informed consent and their risk will be calculated using the FMF tool. Pregnant women with a risk < 1/100 will not be randomized in the study, but will receive the standard follow-up in accordance with the local routine care. Pregnant women with a risk ≥1/100 will be randomized in one of the three study groups.

### Randomization

**Fig. 2:**
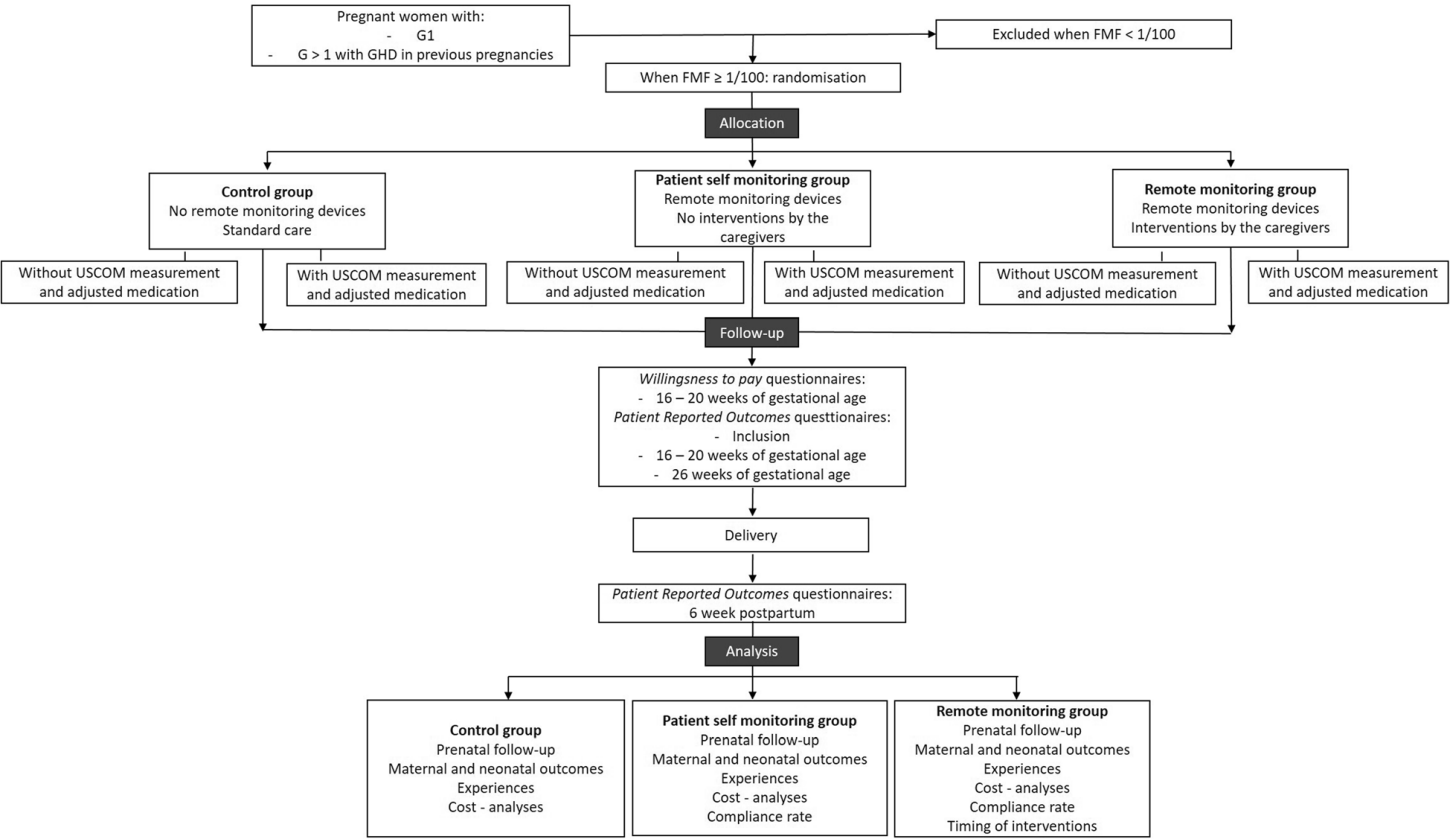
Randomization process and study flow

**Fig. 3:**
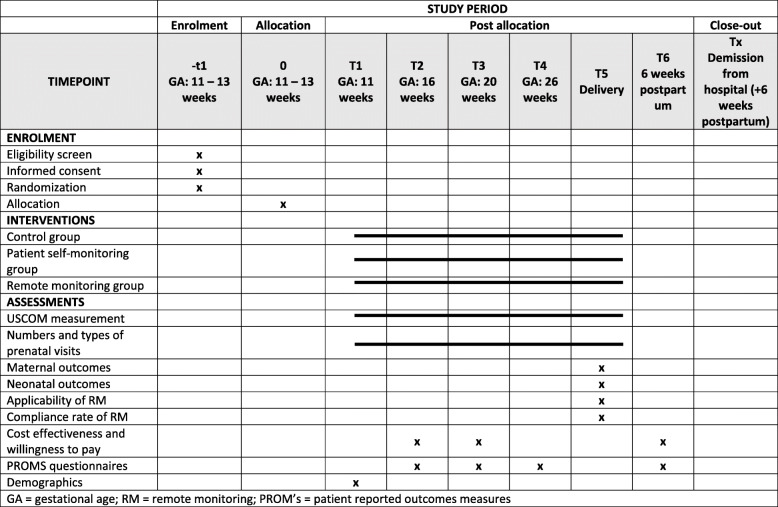
Timeline table – SPIRIT. G = gravidity; FMF = Fetal Medicine Foundation tool

Figures 2 and 3 show the randomization process, trial flow and timeline table (according to SPIRIT). Randomization will be performed between 11 and 14 weeks of gestation, after the first trimester ultrasound examination. Using the Castor EDC web-based system, the women will be randomized to one of the three study groups at a 1:1:1 ratio by the study midwives, who also perform the inclusion in the PREMOM II study and the further RM follow-up (Figs. 2 and 3).

#### Control group (CG)

Women in the CG will not be provided RM devices, and will instead receive standard prenatal care for women at increased risk of developing GHD.

#### Patient self-measurement (PSM) group

Women randomized to the PSM group will collect data, as per the RM group, but their data will not be sent to caregivers at the hospitals and interventions will be performed according to usual care. This group is included as a placebo control group for RM. The pregnant women in this group will be given a blood pressure monitor (BP5, iHealth, Paris, France) and will be asked to measure blood pressure twice daily. They will also record their body weight each week in the app. Only the pregnant women will be able to view the data recorded in the app. They will not be contacted by the study midwife when they aren’t compliant to the study protocol anymore. The compliance rate is a part of the study outcomes. All data will be stored for later use in data analyses.

#### Midwife-assisted RM group

Pregnant women allocated to the midwife-assisted RM group will also receive a blood pressure monitor, as used in the PSM group, and will be asked to measure their blood pressure twice daily. They will also register their body weight every week in the app. The data will be sent to the hospital for review by the allocated midwife. The midwife will contact the responsible obstetrician if any abnormal values are detected allowing interventions to be performed if necessary, or when the pregnant woman doesn’t take her vital parameters for more than 48 h. This study group is identical to that in PREMOM I.

#### Subgroup randomization

The women in all three study groups will be randomized in two subgroups at a 1:1 ratio, as follows.

- Subgroup 1, medication adjustment: If an increase in blood pressure is detected, the woman’s cardiovascular profile will be measured using a hemodynamic monitor (USCOM 1A Uscom, Sydney, Australia). Based on the results, adjusted antihypertensive medications will be given to the woman.
- Subgroup 2, no medication adjustment: when an elevation of the blood pressure happens, the cardiovascular profile will not be measured. The pregnant woman receives the medication following the local obstetrician’s standard procedures.

In total are there six study groups into which the pregnant women can be divided.

### Treatment of pregnant woman

All pregnant women in each group are started on aspirin (Asaflow©), 160 mg once daily, consistent with the latest scientific evidence (e.g., from the Aspirin for Evidence-based Pre-eclampsia Prevention Trial [29]) concerning the follow-up of pregnant women at increased risk of developing PE. Pregnant women allocated to the ‘adjusted medication subgroup’ will be treated according to the cardiac output measured using a USCOM 1A monitor once blood pressure problems are detected. Cardiac output shows three presentations, each of which involves different treatments selected based on recent publications [30–39]. Women with low cardiac output will receive a calcium blocker, women with a normal cardiac output will receive α-methyldopa, and women with high cardiac output will receive a β-blockers. The reference values are shown in Table 1 [40].

**Table 1:** Normal cardiac output values

	Age	16–25	26–35	36–45	45–55	> 55	
**Circulation**	Cardiac output	4.6–7.1	4.8–6.8	4.7–6.7	4.2–5.9	3.5–4.8	l/min

### Outcome measures

#### Primary outcome measures

The primary outcomes measures for the PREMOM II study will be: (1) prenatal follow-up data, including the numbers of prenatal consultations, ultrasounds, cardiotocograms, hospitalizations to a prenatal ward, and duration of hospitalization; (2) delivery data, including duration of labor, complications, mode of delivery, and date of delivery; (3) neonatal data, including gestational age at delivery, date and hour of delivery, APGAR, birth weight, length, complications, NICU hospitalization, and duration of hospitalization; (4) RM data, including duration of participation, number of contacts with the pregnant women regarding vital signs, number of contacts with the responsible obstetrician, numbers of prenatal consultations and hospitalizations due to RM data, and information about the interventions; and (5) compliance rates in the midwife-assisted RM and PSM groups.

#### Secondary outcome measures

Three sets of secondary outcome measures are planned: (1) cost–benefit and willingness to pay analyses to assess health economics; (2) patient-reported outcome measures (PROMs) assessed using questionnaires to document the women’s experiences; and (3) gestational outcomes in relation to administration of antihypertensive drugs.

### Data collection

Data on background variables, obstetric outcomes, and neonatal outcomes after delivery will be collected from the electronic patient files recorded at each hospital. Data on blood pressure and the associated compliance rate will be collected from DHARMA, an online platform developed by the Mobile Health Unit (Limburg Clinical Research Center, Hasselt University – ZOL - Jessa). Information about healthcare costs of pre-, peri-, and postnatal care will be collected from the Intermutualistisch Agentschap (IMA) health insurance database [41]. Information about the PROMs will be recorded using validated questionnaires.

### Sample size calculations

Sample size calculations were performed by Censtat (Hasselt University, L. Bruckers, C. Kremer) to detect clinically meaningful differences between the midwife-assisted RM group and the CG in women with GHD, for two key factors (1) gestational age for neonates born before 34 weeks and (2) admission to the prenatal ward for neonates born after 34 weeks, based on previous work.

1. Gestational age for neonates born before 34 weeks: a difference of ≥10 days between the midwife-assisted RM group and the CG was observed in our pilot studies, and was used as a clinically relevant difference for the power calculation. Priority was given to this sample size calculation because each 1-day increase in gestational age at birth has a significant impact on the neonate’s short- and long-term outcomes
2. Admission to the prenatal ward of neonates born after 34 weeks: a proportional difference of 20% between the midwife-assisted RM group and the CG was considered clinically relevant. This outcome was expected to be most frequent in the PSM group, followed by the CG group, and less likely in the RM group.

The sample size calculations are based on summary statistics derived from our pilot study. A power of 80% and a significance level of 1.67% was used. A within-center variance of 6 (for gestational age) and a random intercept standard deviation of 0.0875 (for prenatal ward admission) was assumed. To obtain 80% power, we estimated that ≥168 GHD pregnancies with delivery before the gestational age of 34 weeks and ≥ 360 GHD pregnancies admitted to the prenatal ward after 34 weeks would need to be included in the study. At each center, the women will be equally divided into the three study groups.

Based on the numbers from Studiecentrum voor Perinatale Epidemiologie (Brussels, Belgium), these 168 births before 34 weeks account for 6.6% of a total of 2545 GHD deliveries. Furthermore, GHD pregnancies represent 4.6% of the total number of pregnancies (55,335) per year [1]. As observed in prior studies, 68% of women with GHD require interventions for their GHD, we estimate a ratio of 100 to 68 pregnant women (or 1.47 to 1) will be needed for the RM group to treat one patient with GHD. As such, 6.76% (4.6% × 1.47) of these women are at risk of developing GHD, meaning 3741 women will need to be enrolled in our study. Taking into account a dropout rate of 4.83% (based on our previous studies), we will need to enroll a total of 3922 women and screen 57,998 women.

For the second sample size calculation (admission to a prenatal ward after 34 weeks), we estimated that > 360 women delivering at a gestational age later than 34 weeks will need to be enrolled across the four prenatal wards. Based on prior studies, ≥42% of women with GHD will be admitted to a prenatal ward. Therefore, out of 5931 deliveries after 34 weeks (6107 eligible women – 176 [168 + dropout rate of 4.83%] who will deliver before 34 weeks), approximately 2491 mothers will be hospitalized. As such, the sample size needed to assess this outcome is expected to be satisfied by the sample size required to show a difference in gestational age in deliveries before 34 weeks.

The primary statistical analysis will entail comparisons among the three study groups using the two-sided Fisher’s exact test at a significance level of 0.05. Adjustment for baseline variables will be performed if necessary, and interaction analyses will be performed using multivariable logistic regression analyses. The median, 95% confidence intervals, quartiles, means, and standard deviation will be calculated as appropriate.

The statistician will be blinded to the study groups.

### Health economics

Cost-effectiveness analyses will be performed, by comparing the healthcare costs between the midwife-assisted RM group, the PSM group, and the CG. Cost-effectiveness analyses are reliable and validated methods for health economic evaluation, and will allow us to estimate whether midwife-assisted RM is desirable relative to current prenatal follow-up. The following aspects will be evaluated in PREMOM II: (1) the incremental cost of midwife-assisted RM to society and (2) the incremental health effects for the pregnant women. The incremental costs will be calculated by comparing the costs of the medical procedures and medications between the CG (women who receive conventional prenatal follow-up) and the midwife-assisted RM group. The difference represents the incremental costs of midwife-assisted RM. Information on healthcare costs will be collected for women in all six study groups from the IMA database. The incremental cost of the health effects will be calculated as the difference in health effects between the CG, PSM group, and midwife-assisted RM group. Relevant health effects will be identified from a validated set of effects using associated questionnaires developed by the International Consortium for Health Outcomes Measurements (ICHOM). We will also assess willingness to pay, by measuring how much pregnant women would like to pay for prenatal follow-up involving RM.

### Stakeholder experiences

In the three study groups, we will use PROMs to map the effectiveness and impact of RM, as estimated by the pregnant woman. The PROMs listed in Table 2 will be included in this study. Each of the PROMs will be measured using a validated questionnaire. Additionally, as PROMs recommended by ICHOM, the investigators will include EQ-5D-5L questionnaires.

**Table 2:** Overview of patient-reported outcomes

PROM	Questionnaire
Quality of Life	EuroQoL EQ-5D-5L
Mental Health	ICHOM (Patient Health Questionnaire 2)
Satisfaction with care	ICHOM
Healthcare responsiveness	ICHOM
Birth experience	ICHOM (Birth Satisfaction Scale – Revised)

The questionnaires listed in Table 2 will be collected via a single questionnaire that will take up to 10 min to complete.

## Status of the study

Recruitment started on September 1, 2019, and enrollment will continue until September 1, 2022. After this time, follow-up will continue for 6 months. The study is at this moment ongoing, patient recruitment is not yet completed at the moment of submission of this manuscript. No other publications containing the results of this study have already published or submitted to any other journal.

## Discussion

The PREMOM II study is the first RCT to evaluate the benefit of RM in the prenatal follow-up of pregnant women at increased risk of GHD. Our previous studies have already indicated that including RM in their care path will result in better pregnancy outcomes as well as a reduction in healthcare costs. Additionally, the stakeholders had positive views of RM. Although the results are promising, it is necessary to confirm them and to identify how RM provides these benefits. To answer these questions, we have designed PREMOM II as a multicenter RCT, in which the timing of interventions will be recorded and a PSM group will be included as a ‘placebo’ group.

Our first hypothesis is that women in the midwife-assisted RM group will have lower numbers of prenatal consultations and unplanned hospital visits compared with the PSM group and the CG. We also believe that women in the PSM group will have the greatest healthcare consumption because their vital signs are not being reviewed by a healthcare professional and no one is able to provide them with specific advice or interpretations, so these women are likely to seek clinical support. Our second hypothesis is that the gestational outcomes will be improved in the midwife-assisted RM group as a consequence of fewer inductions, a greater number of spontaneous deliveries, fewer admissions to an NICU, and fewer diagnoses of PE relative to the CG. We expect that the outcomes of the CG and the PSM group will be the same because the women in these groups are not supervised by a healthcare worker. Related to the previous hypothesis, our next hypothesis is that midwife-assisted RM will not lead to an increase in costs to the healthcare system relative to conventional prenatal care in the CG, and that healthcare costs will be greater in the PSM group than in the CG. These expectations are based on the expected prenatal follow-up and the perinatal outcomes because interventions incur costs and negative gestational outcomes are expensive, and likely to exceed the cost of RM itself. Our penultimate hypothesis is that the number of interventions will be greatest in the midwife-assisted RM group, followed by the PSM group, and the CG. This assumption considers that the timely interventions will result in fewer evolutions of GHD to PE, leading to improved perinatal outcomes in the midwife-assisted RM group. This effect is expected to be smallest in the CG. We also think that appropriate adjustment of antihypertensive medications will result in improved outcomes. Our final hypothesis is that the midwife-assisted RM group will be associated with the most positive stakeholder perceptions, followed by the PSM group. The CG is expected to report the least positive perceptions.

The ultimate goal of this project is to introduce RM into the prenatal care path for women at increased risk of developing GHD throughout Flanders. Implementing RM in every Flemish prenatal ward will ensure nearly all women in this region will have access to this healthcare strategy, improving the maternal and neonatal outcomes. From a global perspective, this RCT will provide a thorough evaluation of the benefit of incorporating RM into the prenatal follow-up of more pregnant women at increased risk of GHD. Successful results of our study will help convince the caregivers, hospital administrators, and the government to incorporate RM into routine clinical practice.

### Project organization

Five centers are participating in the PREMOM II study: the Department of Obstetrics & Gynecology of ZOL (Genk, Belgium), Department of Obstetrics & Gynecology of UZA (Antwerp, Belgium), Department of Obstetrics & Gynecology of Universitaire Ziekenhuis Leuven (Leuven, Belgium), Department of Obstetrics & Gynecology of AZ Sint Jan Brugge–Oostende (Brugge, Belgium), and Department of Obstetrics & Gynecology of AZ Sint Lucas Brugge (Brugge, Belgium). The project has been initiated by the Limburg Clinical Research Center Partners Hasselt University (Hasselt, Belgium) and ZOL (Genk, Belgium), and s being led by the management committee, comprising Prof. Dr. Wilfried Gyselaers, dr. Ir. Inge Thijs, dr. Dorien Lanssens, Prof. Dr. Eric De Jonge, Dr. Caroline Van Holsbeke, Dr. Tinne Mesens, Prof. Dr. Yves Jacquemyn, Prof. Dr. Roland Devlieger, Prof. Dr. Kristel Van Calsteren, Dr. Barbara Lebbe, and Dr. Hilde Logghe. The management committee is responsible for study design, coordination between the centers, the progress of the study, and for the results, which will be analyzed and summarized for publication. The members of the management committee are also the responsible persons of interest, and take responsibility for the project at their individual center. At each center, a midwife will take responsibility for enrollment and follow-up. Prof. Dr. Wim Marneffe and Janis Luyten (groep, unief) will be responsible for analyses of health economics and stakeholder perceptions. Censtat (Hasselt University with Prof. Dr. Liesbeth Bruckers and Cécile Kremer) are providing support for data analysis.

## Data Availability

The datasets used and/or analysed during the current study will be available from the corresponding author on reasonable request.

## Abbreviations

PREMOM: Pregnancy remote monitoring
RM: Remote monitoring
GHD: Gestational hypertension
PROMS: Patient-reported outcome measures
GH: Gestational hypertension
PE: Pre-eclampsia
EH: Essential hypertension
CG: Control group (conventional care)
NICU: Neonatal intensive care unit
FMF: Fetal medicine foundation tool
PSM: Patient self monitoring
IMA: Intermutualistisch agentschap
ICHOM: International consortium for health outcomes measurements
